# News and Coming Events

**Published:** 1907-06-22

**Authors:** 


### NEWS AND COMING EVENTS.

The Queen has sent a present of boxes of chocolate to the Chelsea Hospital for Women.

The Prince and Princess of Wales will visit St. Bartholomew's Hospital on Tuesday, July 23 next, in order to open the new out-patients' department.

The new premises of the City of London Lying-in Hospital, 102 City Road, E.C., will be opened by H.R.H. Princess Christian of Schleswig-Holstein on Monday, July 1 at 3 p.m.

On June 18 £23,500 had been received by the Metropolitan Hospital Sunday Fund, including £1,262 from St. Michael's, Chester Square, through Canon Fleming, the Fund's Golden Pheasant.

The annual anniversary festival dinner of the Metropolitan Hospital, Kingsland Road, N.E., will be held at the Whitehall Rooms, Hôtel Métropole, on Wednesday, July 10, at 7.30 p.m. Mr. Lionel de Rothschild will preside.

The annual meeting of the Association for the Oral Instruction of the Deaf and Dumb will be held at 11 Fitzroy Square, W., on Friday, June 28, 1907, at 3.15. A short illustration of the system will be given, and the President, the Right Hon. the Earl of Crewe, will preside.

The annual report for 1906 of the Medical Officer of Health for the County Borough of Hastings, shows that Hastings holds the record among English towns so far as a low death-rate from epidemic diseases is concerned. During the year the death-rate from zymotic disease was 0.67 per thousand in that town.

Sir George White, presiding over the annual meeting of the Queen Victoria Memorial Hospital, Nice, stated that the total capital outlay on the hospital would be 400,000 francs. Sir Henry Samuelson has borne the cost of the isolation wards, and 15 beds had been endowed from various sources.

The Local Government Board have established new premises for the preparation and distribution of glycerinated calf lymph for the use of public vaccinators, and have notified that all applications for calf lymph should now be addressed to the Government Lymph Establishment, Colindale Avenue, The Hyde, N.W.

The bazaar which the Duchess of Wellington opened at the Royal Hospital for Incurables, Putney Heath, last week, was quite as successful as in former years. The amount realised was just over £550. The authorities state this money does not go into the general funds of the hospital, but is distributed among the 216 in-patients, and serves as pocket money for the ensuing twelve months. The patients are thus able to buy clothing and other personal necessities.

The Dickens Birthplace Fellowship, of which the Mayor of Portsmouth is President, is seeking to raise a permanent memorial to Charles Dickens in his native town, by endowing a cot in the hospital situated in the parish in which he was born. This hospital is The Royal Portsmouth and Gosport Hospital, and the cot cost £500. The first £100 is already assured, and among the subscribers are some of the most distinguished names in the country. All admirers of Charles Dickens are appealed to in aid of this good work.

The fourteenth International Congress of Hygiene and Demography will be held in Berlin from September 23 to 29 next. The committee invites the attendance of practitioners from all countries.

The Garden Fête and Sale of Work in aid of the funds of the Hospital and Home for Incurable Children, Northcourt, College Villas Road, Hampstead, N.W. (near Finchley Road and Swiss Cottage Stations), which was to have been held on Saturday, June 29, 1907, has been postponed owing to a slight outbreak of chicken-pox in the Home.

The sale by Messrs. Phillips, Son and Neale, of 23 New Bond Street, W., of the furniture and effects of the late Mr. George Herring, which are to be disposed of under his will for the benefit of King Edward's Hospital Fund for London, will take place at 1 Hamilton Place, Park Lane, W., on Wednesday, June 26, and the two following days. There will be some six hundred lots, including the handsome appointments of several rooms, and many valuable cabinets, sculpture, and china. The sale has a special interest for hospital people, and is likely to be largely attended.

The judicial authorities at Versailles have announced that there is no charge whatever against Dr. Hebert, so that his character is without a stain. "I had considered all along" (writes the Paris correspondent of the *Daily Telegraph*) "that there was no evidence whatever against him, and it seems very hard that he should have been detained so long in custody, when, moreover, he was suffering cruelly from the effects of the murderous assault perpetrated on him at Bois-le-Roi, and should have been subjected to so many unkind remarks and criticisms. The trial of Madame Guérin is now fixed to take place at the Versailles Police Court, on July 11, and Dr. Hebert will, as is understood, lay claim to damages."

Dr. J. S. Milne has now completed a long monograph on "Surgical Instruments in Greek and Roman Times," which will be issued from the Oxford University Press within a few days. No clear conception of a surgical operation, ancient or modern, can be formed from a written description without some previous knowledge of the instruments, and the author points out that many interesting operations described in detail in the clinical authors are rendered obscure or quite unintelligible from lack of such knowledge. No systematic attempt to reconstruct the different instruments used by the ancients has hitherto been made, this department of archæology having received scant attention. The volume, which embodies investigations extending over several years, is illustrated.

In common with other Eastern countries, Korea has thousands of lepers. The Mission to Lepers in India and the East hopes to provide an asylum for some of them in the neighbourhood of Fusan. At present they subsist on casual alms, and in cold weather crawl into the fireplaces for warmth, being practically naked, and specially sensitive to cold. "They are" (writes the Rev. W. E. Smith) "truly outcasts, despised and shunned of all men." Land and houses are cheap, and, once founded, the colony would be nearly self-supporting. The society's funds are sorely taxed for the maintenance of the fifty asylums in India, China, and Japan for which it is already responsible. Gifts towards this new effort for Korean lepers would be gratefully received by the Organising Secretary, Mr. John Jackson, F.R.G.S., at Exeter Hall, Strand.

